# Altered Lung Microbiome and Metabolome Profile in Children With Pulmonary Arterial Hypertension Associated With Congenital Heart Disease

**DOI:** 10.3389/fmed.2022.940784

**Published:** 2022-07-28

**Authors:** Runwei Ma, Liming Cheng, Yi Song, Yi Sun, Wenting Gui, Yao Deng, Chao Xie, Min Liu

**Affiliations:** ^1^Department of Cardiovascular Surgery, Fuwai Yunnan Cardiovascular Hospital, Kunming, China; ^2^Department of Anesthesiology, Kunming Children's Hospital, Kunming, China; ^3^Department of Extracorporeal Circulation, Fuwai Yunnan Cardiovascular Hospital, Kunming, China

**Keywords:** pulmonary arterial hypertension, congenital heart disease, left to right shunt, lung, microbiome, metabolome

## Abstract

**Backgrounds:**

Pulmonary arterial hypertension (PAH) is characterized by progressive pulmonary vascular functional and structural changes, resulting in increased pulmonary vascular resistance and eventually right heart failure and death. Congenital Left-to-Right shunts (LTRS) is one type of congenital heart disease (CHD) and PAH associated with the congenital Left-to-Right shunt (PAH-LTRS) is a severe disease in children. However, changes in the lung microbiome and their potential impact on PAH-LTRS have not been not fully studied. We hypothesized that lung microbiota and their derived metabolites have been disturbed in children with PAH-LTRS, which might contribute to the progression and outcomes of PAH-LTRS.

**Methods:**

In this study, 68 age- and sex-matched children of three different groups (patients with PAH-LTRS cohort, patients with LTRS but have no pathologic features of PAH cohort, and healthy reference cohort) were enrolled in the current study. Bronchoalveolar lavage fluid samples from these participants were conducted for multi-omics analysis, including 16S rRNA sequencing and metabolomic profiling. Data progressing and integration analysis were performed to identify pulmonary microbial and metabolic characteristics of PAH-LTRS in children.

**Results:**

We found that microbial community density was not significantly altered in PAH-LTRS based on α-diversity analysis. Microbial composition analysis indicated phylum of Bacteroidetes was that less abundant while Lactobacillus, Alicycliphilus, and Parapusillimonas were significantly altered and might contribute to PAH in children with LTRS. Moreover, metabolome profiling data showed that metabolites involved in Purine metabolism, Glycerophospholipid metabolism, Galactose metabolism, and Pyrimidine metabolism were also significantly disturbed in the PAH-LTRS cohort. Correlation analysis between microbes and metabolites indicated that alterations in the microbial composition from the lung microbiota could eventually result in the disturbance in certain metabolites, and might finally contribute to the pathology of PAH-LTRS.

**Conclusion:**

Lung microbial density was not significantly altered in patients with PAH-LTRS. Composition analysis results showed that the relative microbiome abundance was different between groups. Metabolome profiling and correlation analysis with microbiota showed that metabolome also altered in children with PAH-LTRS. This study indicated that pulmonary microbes and metabolites disturbed in PAH-LTRS could be potentially effective biomarkers and provides valuable perspectives on clinical diagnosis, treatment, and prognosis of pediatric PAH-LTRS.

## Introduction

PAH is a multifactorial disease characterized by progressive loss and obstructive remodeling of the pulmonary arteries (1). The disease leads to elevated pulmonary vascular resistance (PVR) together with pulmonary arterial pressure (mPAP), ultimately resulting in irreversible right ventricular failure and death (2–5), and median survival in patients with PAH is only 5–7 yr (6). Although significant efforts have been made in the last decades, PAH is still incurable and needs life-long treatments (7), significantly influencing morbidity and mortality. The 6th World Symposium on pulmonary hypertension modified the definition for pulmonary hypertension as mPAP > 20 mmHg and PVR ≥ 3 Wood Units (WU) instead of only mPAP > 25-mmHg (3).

PAH is a complication widely found in patients with various congenital heart diseases (CHD) (8), including LTRS (9, 10). LTRS contributes to pulmonary vasculature remodeling and increased PVR, ultimately leading to PAH. Pediatric PAH has similar characteristics to adults but not the same in treatment and outcomes (11). Pediatric cardiovascular medicine and surgery still were the main therapeutic strategies for these patients (12). Correction is critical to preventing pulmonary vascular remodeling and progression of PAH-LTRS. If not, it might ultimately develop into Eisenmenger's syndrome, the most severe phenotype of PAH associated with CHD (13–15). For patients with end-stage PAH, lung function is severely disrupted, and difficulty in breathing. In that case, although lung transplantation was the only established treatment option (16–18), organ rejection and infection will be the most significant challenge after surgery. Early diagnosis or early treatment of LTRS or PAH-LTRS will effectively reduce the progression and mortality rate of PAH (19). Closure of cardiac defects in early childhood could effectively prevent the occurrence and progression of PAH and has a favorable outcome (20). However, not all children can be repaired timely and successfully, especially in developing countries (20). For them, they may continue to suffer from PAH their whole life. Although children with PAH-LTRS performed atrial septal defect closure at an appropriate time, most might persist immediately or initiate PAH months or years later despite the lack of any residual shunt named post-operation PAH. Postoperative PAH has the poorest prognosis, even worse than uncorrected subjects or Eisenmenger syndrome (21), significantly influencing the survival.

Although there have been notable advances in therapies for PAH, there are still considerable challenges in the cure and prognosis of the disease, specifically associated with CHD. Physical activity, pathology, and biomarkers are used for prognostic evaluation of PAH treatment in children but are far from sufficient to treat (22–25). Therefore, new treatment strategies or diagnostic biomarkers for PAH associated with CHD are urgently required in therapies or prognoses in the future. Microbiota facilitates many physiological functions by affecting host systemic immune regulation, energy homeostasis and metabolism, vitamin synthesis and degradation, and is widely considered involved in various diseases (26–30). Gut microbiota dysbiosis in patients with PAH was observed by extensive studies (31–34). Gut microbiota-derived metabolites were also involved in PAH through effects on the gut-lung axis (35, 36).

The lung is similar to the gut in immunity, epithelial barrier functions, and microbiomes. The microbiome in pulmonary is also the source of clinical variation in critical illness (37–39), despite the biomass of lung microbiota being smaller than the gut. The lung microbiome and metabolome could contribute to respiratory infections and inflammation, relating to human health and resulting in various pulmonary diseases, and could be the novel therapeutic target for the prevention and treatment or predicting clinical outcomes of human diseases (40–42). Knowledge of pulmonary microbiota and their derived metabolites have not been fully characterized in patients with PAH, especially in patients with PAH-LTRS. Lung is the local tissue where PAH occurs and severe PAH in patients with LTRS eventually results in lung transplantation without treatment (18). Therefore, it seems reasonable to assume that lung microbiome and metabolome have changed in patients with PAH-LTRS. Identification and characterization of lung-specific bacteria and metabolites in PAH-LTRS could lead to the development of targeted therapies in the future.

With the emerging role of the microbiome in disease, lung microbiota composition and function were potentially crucial for diagnosis, treatment, and improving clinical outcomes for patients with PAH-LTRS. Thus, our objective in this study was to evaluate the hypothesis that children with PAH-LTRS have a unique microbiome and metabolome profile in the lung that potentially, in turn, contribute to the pathogenesis and outcomes of PAH. Our results demonstrate that the lung microbiome was not changed globally but significantly altered in some taxa, in terms of composition at phylum and genus levels. These disturbances might contribute to the alteration of metabolites and might contribute to the pathology of PAH-LTRS.

## Materials and Methods

### Patients and Bronchoalveolar Lavage Fluid Collection

Patients who underwent clinical bronchoscopy at the Fuwai Yunnan Cardiovascular Hospital (Kunming, China) were recruited in our study. The pulmonary microbial community was easily affected by multi-factors and subjects were excluded from our study if they (1) have other cardiopulmonary diseases, lung comorbidities, pulmonary infection, metabolic diseases, and other systemic diseases; (2) take any special drugs, such as antibiotics, in the 3 months before enrollment; (3) have special diets such as feeding with breast or formula; and (4) participate in other clinical studies or were unable to provide consent for care or BALF collection. Finally, a total of 68 patients were enrolled in the current research. Bronchoalveolar lavage fluid (BALF) was collected into the sterile tube according to a standardized protocol with minimum oral contamination (43). Two milliliter BALF was collected from each participant and flash-frozen in liquid nitrogen and transferred to −80°C later until further processing.

### 16S rRNA High-Throughput Sequencing and Data Processing

Bacterial genomic DNA was extracted from BALF samples with CTAB/SDS method according to the previously reported (44). 16S rRNA high-throughput sequencing was performed with the 16S MetaVx™ system (GENEWIZ). Total genomic DNA with ~3 ng was conducted to first step PCR to amplify 16S rRNA genes (V3–V4) as per manufacturer's instructions. Purified PCR products were processed for second step PCR to construct sequencing libraries. 16S rRNA was sequenced on the Illumina MiSeq platform, and paired-end reads were obtained. Data processing according to previously described (34). In summary, raw data was firstly performed to eliminate adaptors by Cutadapt (1.9.1), and then input into Vsearch (1.9.6) and Qiime (1.9.1) for further filtration and operational taxonomic units (OUTs) analysis. Alpha diversity indexes, including observed species, Chao1, Shannon, and PD whole tree, evaluated by Qiime, were employed to analyze the complexity of bacterial diversity, and the *P*-values were adjusted by the Benjamini-Hochberg correction. Unsupervised analysis methods, such as principal coordinate analyses (PCoA), could significantly detect the difference between groups. However, when the differences between the groups are not significantly different and the obvious differences existed within groups, unsupervised analysis is difficult to find and distinguish the differences between the groups. Partial least squares-discriminant analysis (PLS-DA), as the supervised method, could achieve dimensionality reduction for clustering but with full awareness of the group labels and was also performed to analyze the potential differences among different groups in our study. Online data processing tool LEfSe (http://huttenhower.sph.harvard.edu/galaxy/root?tool_id=lefse_upload) was used to find dominant taxa in each group. Wilcoxon rank sum test was performed to evaluate taxonomic abundance in different groups. If not specified, figures were drawn by R packages.

### Metabolomic Profiling by Liquid Chromatography and Mass Spectrometry and Data Processing

BALF samples used for metabolomic assay were taken from liquid nitrogen and thawed on ice, and metabolite extraction was performed using methanol and L-2-chlorobenzalanine. Metabolomic analysis of all samples was performed using the UHPLC -Q Exactive HF-X system (Thermo). The instrument was equipped with an ACQUITY UPLC HSS T3 (100 mm × 2.1 mm i.d., 1.8 μm; Waters, Milford, USA), and the column temperature was maintained at 40°C. Gradient elution of analytes was performed as previously described (45). Quality control samples are prepared by mixing equal volumes of all assay samples and are used to evaluate assay system stability. Obtained metabolism data were progressed according to previously reported (46). Briefly, Principal Component Analysis (PCA) was conducted to analyze global similarities containing quality control samples (QC). Student's *t*-test combined with multivariate analysis Orthogonal partial least-squares discriminate analysis (OPLS-DA) method was utilized to screen out the differential metabolites between groups (VIP > 1, *p*-value <0.05). Differential metabolites (DMs) were performed based on fisher's exact test and clustering of DMs according to the Pearson coefficient. Differentially enriched KEGG (Kyoto Encyclopedia of Genes and Genomes) was performed to investigate the metabolomic pathways potentially involved in the pathogenesis of PAH-LTRS pathways by integrating databases of HMDB, KEGG compound and LIPID MAPS.

### Correlation Between the Lung Microbiota and the Metabolome

Pearson correlation analysis was performed to reveal the correlation between lung microbiota and the metabolites with the Cytoscape software as previously described (47). *P* < 0.05 was regarded as statistically significant, *P* < 0.01 was considered as very significant, and *P* < 0.001 was regarded as extremely significant. A heatmap was used to show the correlation between lung bacteria and metabolites.

### Statistical Analysis

All data were shown as the mean ± standard error of mean (s.e.m.). Patient's baseline characters and F/B ratio were graphically plotted using GraphPad Prism 7 (GraphPad Software Inc. San Diego, CA, USA). One-way ANOVA was used for assessing differences among multiple groups. Differences were considered statistically significant at *p* < 0.05.

## Results

### Study Populations

Six-eight anticipants, in three cohorts [21 children with PAH-LTRS cohort, 26 children with only LTRS cohort, and 21 healthy reference (REF) cohort] were recruited in our current study. The average age of these participants was 3.377 years, and the proportion of males and females was 60.3 and 39.7%, respectively. There were no significant differences in age and sex among the three groups. Baseline characteristics of anticipants are shown in Table 1.

**Table 1:** Characteristics of participants.

**Characteristic**	**All patients (*N* = 68)**
Mean age (s.e.m.), yr	3.377 (0.411)
**Sex**
Male, *n* (%)	41 (60.3)
Female, *n* (%)	27 (39.7)
**Groups**
LTRS, *n* (%)	26 (38.2)
PAH-LTRS, *n* (%)	21 (30.9)
REF, *n* (%)	21 (30.9)
**Diagnosis**
VSD, *n*	19
ASD, *n*	9
VSD + PFO, *n*	12
VSD + ASD, *n*	5
VSD + PDA, *n*	1
VSD + PDA + PFO, *n*	1

*Values are mean ± s.e.m., proportiinton*.

*LTRS, congenital left to right shunts heart disease; PAH-LTRS, Pulmonary arterial hypertension associated with the congenital left to right shunts heart disease; REF, healthy reference; VSD, Ventricular Septal defect; ASD, Atrial Septal Defect; PFO, Patent Foramen Ovale; PDA, patent ductus arteriosus*.

BALF specimens were obtained from three cohorts as our study objects (*n* = 68) and were conducted for microbiome and metabolome profiling. Since some BLAF specimens were not sufficient for both analyses at the same time, of these 68 samples, 47 dispersed in different groups were used for 16S rRNA sequencing, while 59 were conducted for metabolomics profiling. All PAH-LTRS participants met the clinical definition of PAH, with exceeding mean PAP 20 mmHg and PVR > 3 Wood units measured by right heart catheterization.

### Bacterial Diversity and Community Structure Were Not Significantly Disturbed in Children With PAH-LTRS

**Figure 1:**
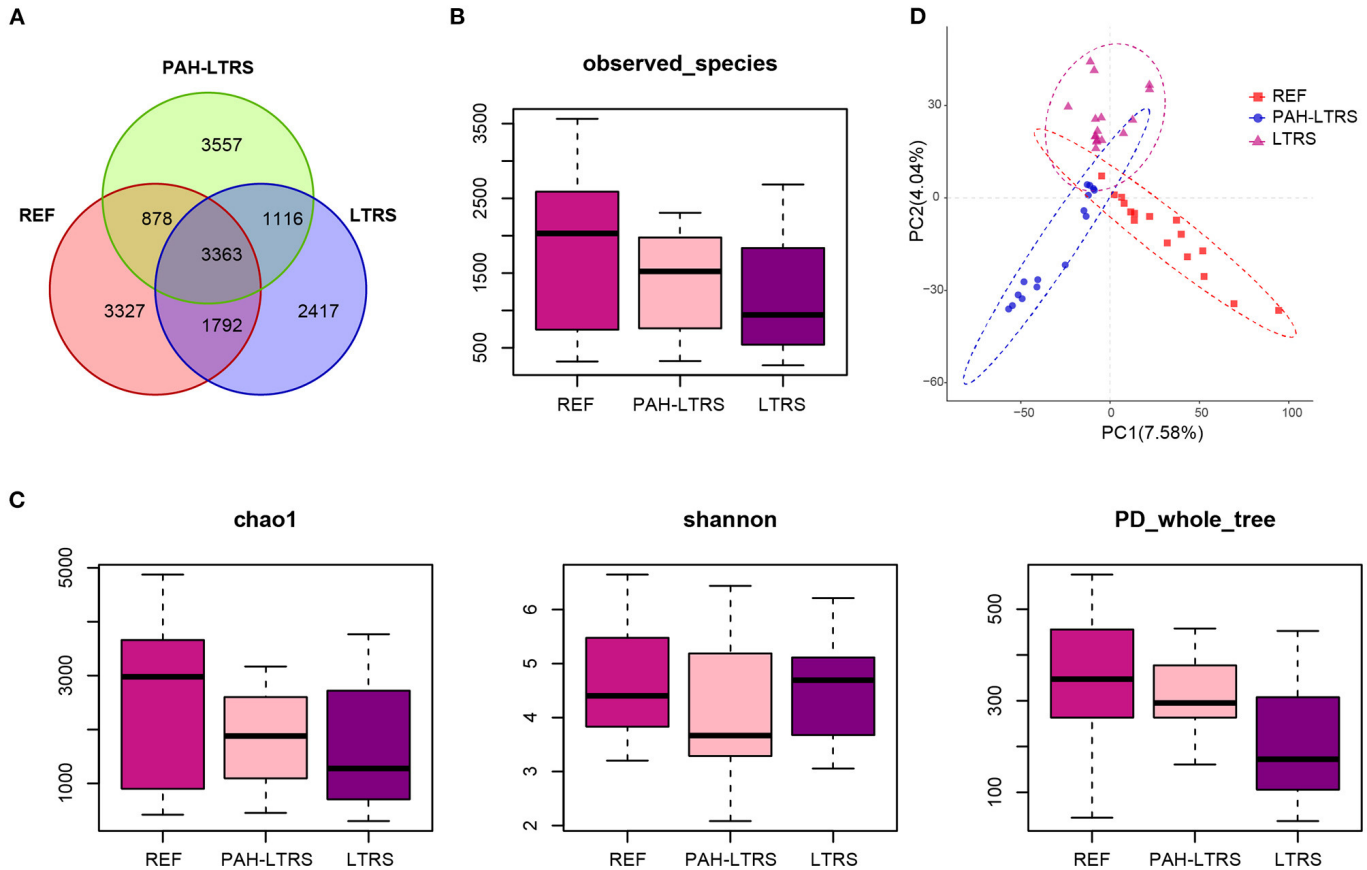
Pulmonary bacterial community diversity in different cohorts and Principal Coordinate Analysis (PCoA) clustering. **(A)** Venn diagram of OUTs detected in three cohorts. **(B)** The number of species identified in three groups. **(C)** α-diversity among three cohorts measured by the Chao, Shannon and PD whole tree. Results were present as means ± s.e.m. **(D)** PLS-DA was conducted to compare the overall similarities in the bacterial taxonomy based on β-diversity. Each principal component represents most of the variation between samples. PAH-LTRS, patients with pulmonary arterial hypertension associated with Left-to-Right shunt (*n* = 15); REF, healthy reference (*n* = 16); LTRS, patients with Left-to-Right shunts but without PAH (*n* = 16).

We firstly analyzed bacterial diversity in each cohort. A mass of operational taxonomic units (OTUs) was identified in our study (Supplementary Table 1), with common and distinct OTUs among different cohorts (Figure 1A). The number of species detected in PAH-LTRS and LTRS patients was slightly lower than that in REF but with no significant differences (PAH-LTRS vs. REF with padj = 0.43, LTRS vs. REF with padj = 0.16, and LTRS vs. PAH-LTRS with padj = 0.83) (Figure 1B). α-diversity analysis was then conducted to evaluate the richness and evenness of species diversity within each cohort. The result showed that PD whole tree index decreased in LTRS compared with REF and PAH-LTRS (padj = 0.005, 0.043, respectively). However, community diversity was not significantly altered globally among these cohorts indicated by the indexes of Chao1 (PAH-LTRS vs. REF with padj = 0.39, LTRS vs. REF with padj = 0.16 and LTRS vs. PAH-LTRS with padj = 0.86) and Shannon (PAH-LTRS vs. REF with padj = 0.39, LTRS vs. REF with padj = 0.99 and LTRS vs. PAH-LTRS with padj = 0.45) (Figure 1C). These results showed that microbiota community diversity was not significantly altered among different cohorts.

To further assess the similarities of bacterial communities among groups, PCoA plot based on the bray-curtis distance (Supplementary Figure 1A) were performed at the OTUs levels based on β-diversity analysis. The PCoA results showed no separation of the PAH-LTRS patients from other groups, indicating that the main composition of the lung microbiome of the PAH-LTRS cohort was not significantly altered. Supervised PLS-DA was then considered to find and distinguish the possible differences in OTUs between the groups and there was discernable boundaries among different cohorts (Figure 1D). Taken together, the results demonstrated that the microbial community in patients with PAH-LTRS was not globally different from other groups.

### PAH-LTRS Patients Have Distinct Lung Microbiome Composition

**Figure 2:**
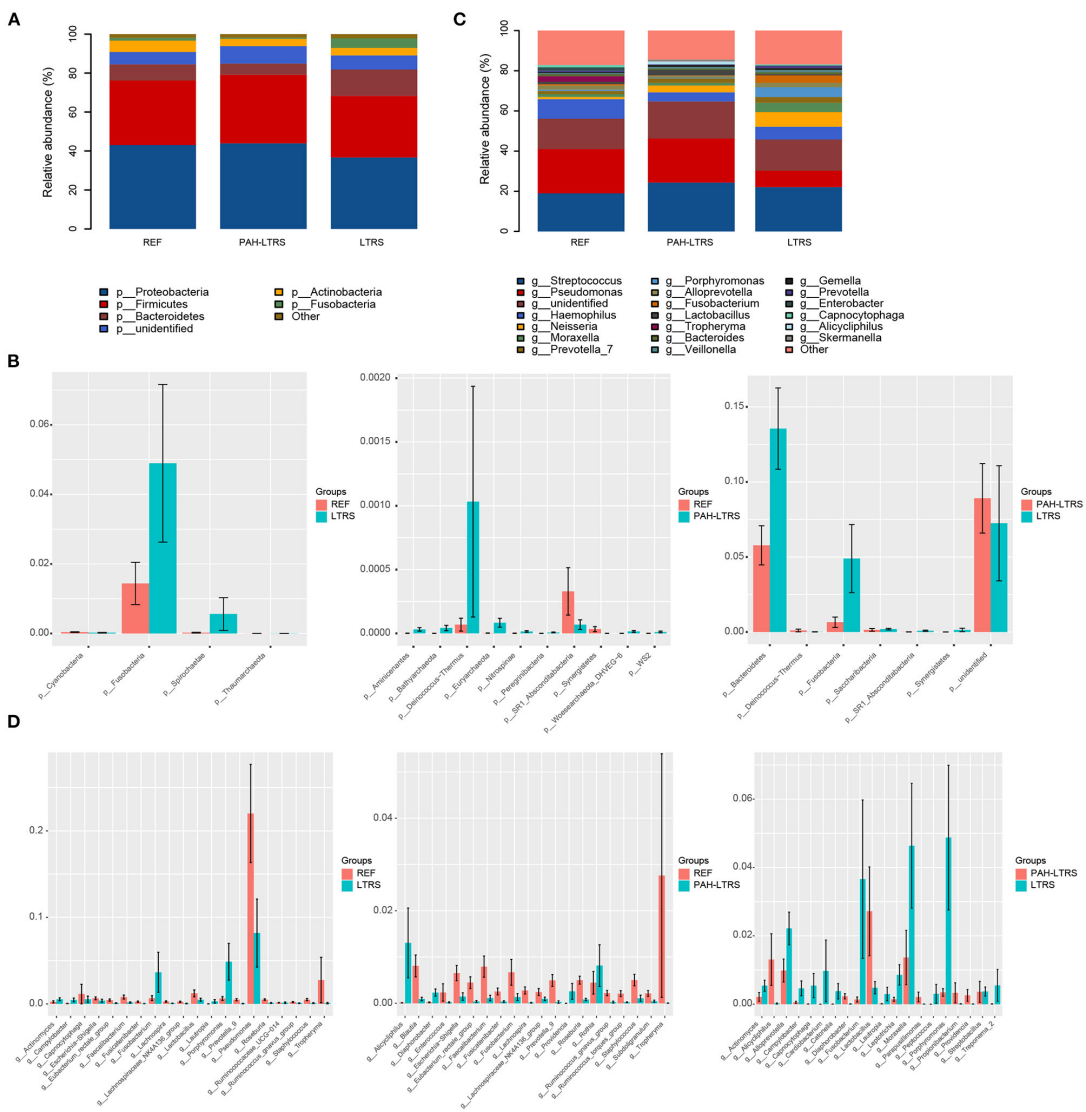
Taxonomic composition of the lung microbiota among three cohorts. **(A)** The relative frequency of top abundant taxa in each cohort at the phylum level. **(B)** The relative abundance analysis of top altered phylum in three pair of comparisons (means ± s.e.m.). **(C)** The relative frequency of top abundant taxa in each cohort at the genus level. **(D)** Analysis of the relative abundance of top fluctuated genera between any two different cohorts (means ± s.e.m.). Wilcoxon rank sum test, *p* < 0.05. PAH-LTRS, patients with pulmonary arterial hypertension associated with Left-to-Right shunt (*n* = 15); REF, healthy reference (*n* = 16); LTRS, patients with Left-to-Right shunts but without PAH (*n* = 16).

To determine the differences among the three cohorts, we next focused on the bacteria composition, which contributes to the microbiota ecosystem. The predominant phyla in three cohorts were Proteobacteria, Firmicutes, Bacteroidetes, Actinobacteria, and Fusobacteria (Figure 2A; Supplementary Table 2). Although many taxa were common among the three subjects, the relative abundance was not the same. In PAH-LTRS, Bacteroidetes (5.77 vs. 8.3% and 13.55%) and Fusobacteria (0.65 vs. 1.43% and 4.8%) were decreased, Compared with REF and LTRS groups while the Bacteroidetes was one of the most important composition contributed to gut microbial homeostasis. Furthermore, we conducted a differential analysis of the relative abundance components between different groups based on the Wilcoxon rank sum test. The proportion of Deinococcus–Thermus and Euryarchaeota were significantly increased (*p* = 0.002, 0.047, respectively) in PAH-LTRS compared with REF (Figure 2B, middle; Supplementary Table S2). Bacteroidetes and Fusobacteria were significantly decreased (*p* = 0.014, 9.12E-05, respectively) in PAH-LTRS compared with LTRS (Figure 2B, right; Supplementary Table 2).

At genus level, the most common bacteria in three groups included Streptococcus, Pseudomonas, Haemophilus, Neisseria and Moraxella (Figure 2C; Supplementary Table 2). We found that the proportion of Haemophilus (4.56 vs. 9.69% and 6.33%) decreased, while Lactobacillus increased in PAH-LTRS compared with REF (2.71 vs. 1.22%) and LTRS (2.71 vs. 0.48%) groups. Compared with REF, among the top 20 most variable genera, Alicycliphilus (*p* = 0.01), Rothia (*p* = 0.03), Providencia (*p* = 0.001) and Diaphorobacter (*p* = 0.006) were significantly increased, while Ruminococcus_gnavus_group (*p* = 6.68E-05), Fusicatenibacter (*p* = 0.001) and Enterococcus (*p* = 0.02) tend to be decreased in PAH-LTRS group (Figure 2D, middle; Supplementary Table 2). Compared with LTRS group, Moraxella (*p* = 0.02), Porphyromonas (*p* = 0.003), Campylobacter (*p* = 0.003) and Fusicatenibacter (*p* = 0.002) were significantly decreased while Alicycliphilus (*p* = 0.004) and Lactobacillus (*p* = 0.039) were increased in PAH-LTRS group (Figure 2D, right; Supplementary Table 2). Taxa composition at phylum and genus levels in each sample were showed in Supplementary Figures 1B,D.

**Figure 3:**
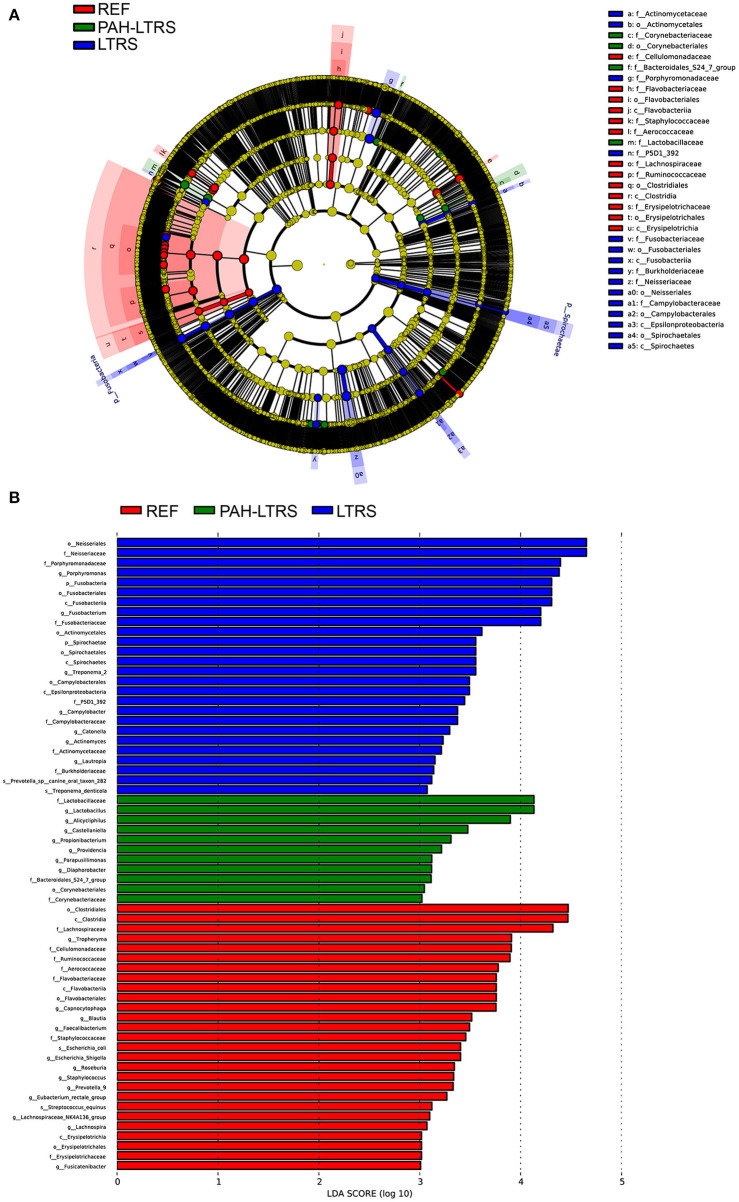
LEfSe analysis to visualize differences in bacterial taxa among different groups. **(A)** The phylogenetic distribution of significantly enriched bacteria in different groups were showed by the cladogram. Nodes with different color represent different microbial taxa that are significantly abundant in the groups and significantly contribute to the differences between cohorts. **(B)** LDA bar graphs were utilized to identify the microbiota taxa with potentially significant effects in the three groups. Linear discriminant analysis (LDA, score > 2.0) scores are shown on the x-axis and taxa with the larger LDA score, the greater effect they might have in this group. PAH-LTRS, patients with pulmonary arterial hypertension associated with Left-to-Right shunt (*n* = 15); REF, healthy reference (*n* = 16); LTRS, patients with Left-to-Right shunts but without PAH (*n* = 16).

LEfSe tool was considered to analyze bacterial communities in BALF samples and to find potentially essential differential taxa. The cladogram shows that Lactobacillaceae, acteroidales_S24-7_group, and Lactobacillaceae families were the predominant taxa and potentially involved in the pathology of PAH-LTRS (Figure 3A). Furthermore, the enriched genera in the PAH-LTRS group included Lactobacillus, Alicycliphilus, Castellaniella, Propionibacterium, Providencia, Parapusillimonas and Diaphorobacter (with LDA threshold 3) (Figure 3B). Collectively, these observations suggested composition of lung microbiota had been altered in PAH-LTRS, leading to dysbiosis in the pulmonary microenvironment and the Lactobacillus genus might have an essential influence on PAH-LTRS.

### The Lung Metabolome Is Altered in PAH-LTRS

**Figure 4:**
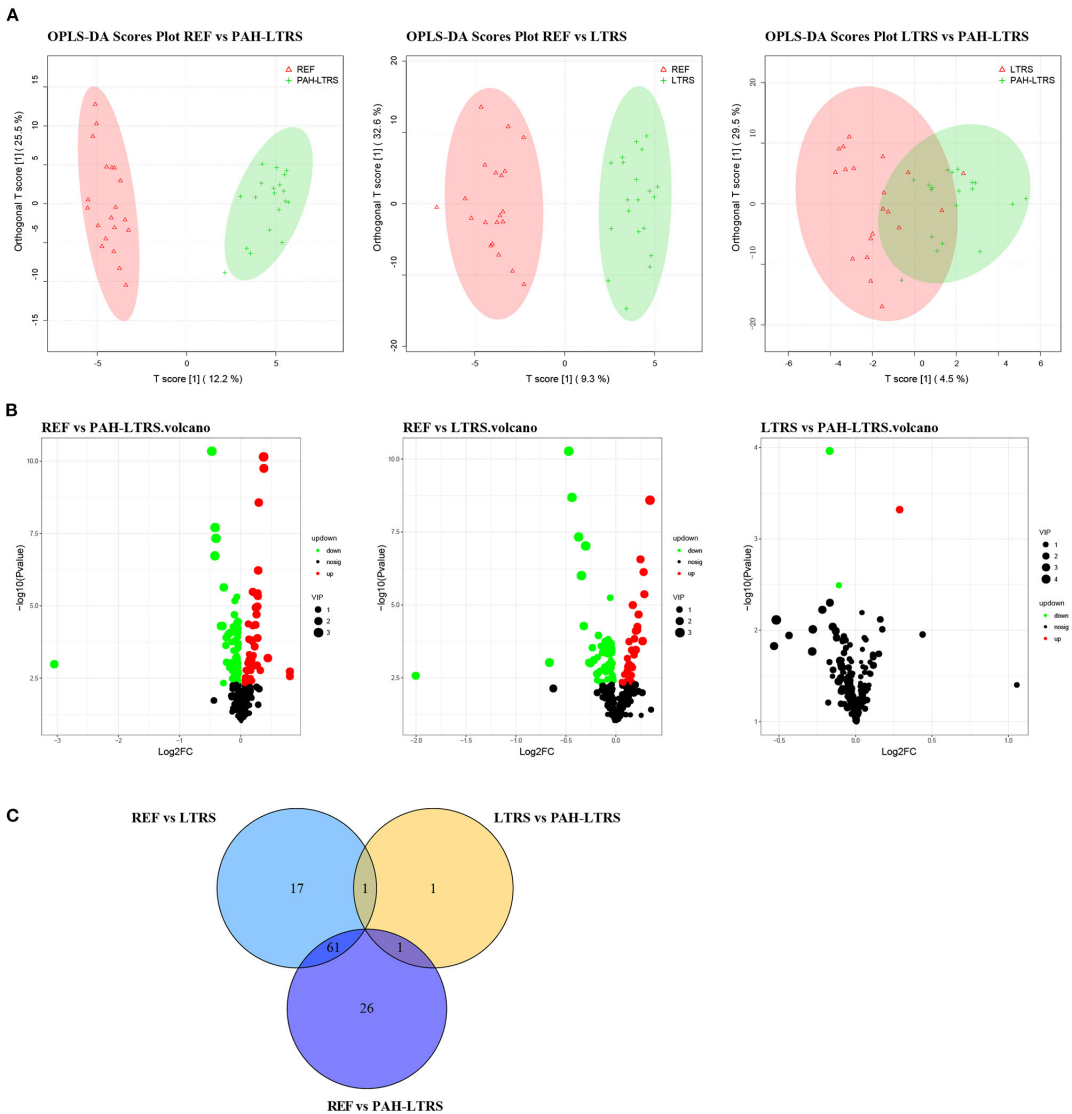
Differential lung metabolites between different groups. **(A)** OPLS-DA was used to discriminate between different groups. **(B)** volcano plot of the differential metabolisms for any two groups (VIP > 1, *P* < 0.05). **(C)** Venn diagram showed the altered metabolites overlap among three pair of comparisions. PAH-LTRS, patients with pulmonary arterial hypertension associated with Left-to-Right shunt (*n* = 19); REF, healthy reference (*n* = 20); LTRS, patients with Left-to-Right shunts but without PAH (*n* = 20).

Accumulating evidence shows that PAH is a systemic metabolic disease. To explore the metabolites that contribute to the pulmonary microenvironment, we then conducted a non-targeted metabolism profiling on a total of 59 BALF samples. PCA analysis containing QC samples was shown in Supplementary Figure 2A. OPLS-DA analysis between any two of three groups showed clear boundaries, despite LTRS and PAH-LTRS were not completely separated in the clustering (Figure 4A). Differential analysis was then performed between groups, and the significantly altered metabolites were selected based on the variable importance in the projection (VIP) values and *p*-values (VIP > 1 and *p* < 0.05). Under this criterion, 88, 3, and 79 metabolites were found to be disturbed in REF vs. PAH-LTRS, REF vs. LTRS, and LTRS vs. PAH-LTRS, respectively (Figures 4B,C). Detailed information on these differential metabolites is available in Supplementary Table 3.

**Figure 5:**
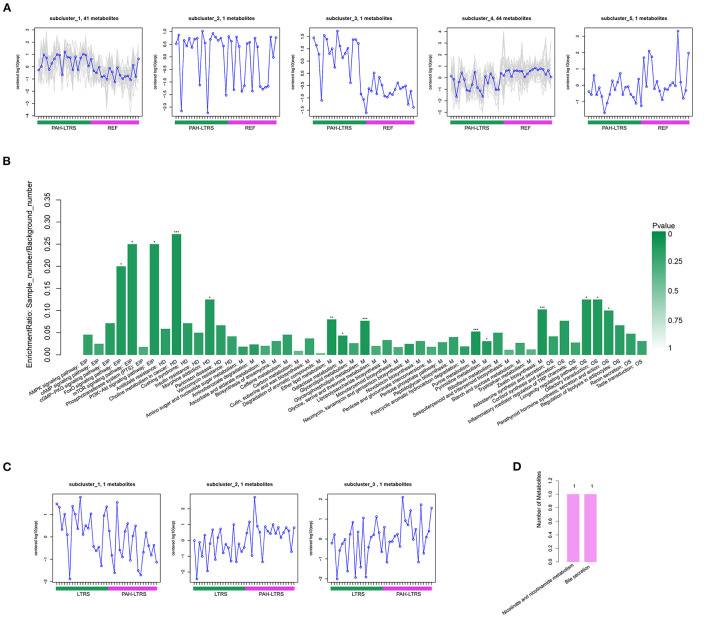
Differential metabolites clustering and KEGG enrichment. **(A)** Differential metabolites in REF vs. PAH-LTRS were clustered into 5 clusters. These sub-clusters had different expression trend in two groups. **(B)** KEGG enrichment of these differential metabolites in REF vs. PAH. **(C)** Three differential metabolites in LTRS vs. PAH-LTRS also shown with different abundance in two groups. **(D)** Three differential metabolites in LTRS vs. PAH-LTRS were annotated into two pathways without significantly enrichment. EIP, Environmental Information Processing; HD, Human Diseases; M, Metabolism; OS, Organismal Systems. PAH-LTRS, patients with pulmonary arterial hypertension associated with Left-to-Right shunt (*n* = 19); REF, healthy reference (*n* = 20); LTRS, patients with Left-to-Right shunts but without PAH (*n* = 20).

In the comparison of PAH-LTRS and REF, the most differential metabolites were identified and clustered into 5 clusters (Supplementary Figure 2B) with different expressed tendencies (Figure 5A). The significantly altered metabolites included Pos-22909 (2-piperidone), neg_1486 (UDP-glucose), neg_15140 (Glycerophosphocholine), neg_532 (ADP-ribose), neg_3243 (Adenosine monophosphate), pos_25479 (Dimethylethanolamine), neg_3243 (Adenosine monophosphate), and pos_147 (Uridine). To further identify the biological significance associated with PAH-LTRS, we applied the Kyoto Encyclopedia of Genes and Genomes (KEGG) database to focus on the discrepant KEGG annotation with differential metabolites was firstly performed (Supplementary Figure 2C), and the significantly enriched terms (with the metabolic pathways most affected) included FoXO signaling pathway, mTOR signaling pathway, PI3K-Akt signaling pathway, Eher lipid metabolism, Choline metabolism in cancer, Galactose metabolism, Glycerophospholipid metabolism, Purine metabolism, Pyrimidine metabolism and Zeatin biosynthesis (Figure 5B). Other classical signal pathways involved in PAH, such as the cGMP-PKG signaling pathway and Insulin resistance, were also dysregulated in PAH-LTRS, although not significantly (*P* = 0.06, 0.08, respectively).

Furthermore, there were only three differential metabolites were found in LTRS vs. PAH-LTRS, 2-piperidone, Pos-25441 (Trigonellinamide) and Pos-18930 (DL-2-Aminooctanoic acid). 2-piperidone was significantly decreased in PAH-LTRS (Figure 5C; Supplementary Figure 2D). These differential metabolites were annotated to Nicotinate and nicotinamide metabolism and bile secretion (Figure 5D). Overall, patients with PAH-LRTS exhibited a distinct metabolic signature compared with that in REF or the LRTS group. These changes in metabolites and related KEGG pathways might both contribute to the alteration of the lung microenvironment in patients with PAH-LTRS.

### Microbes and Their Derived Metabolites Can Be Biomarkers for Targeted Therapy of PAH-LTRS

**Figure 6:**
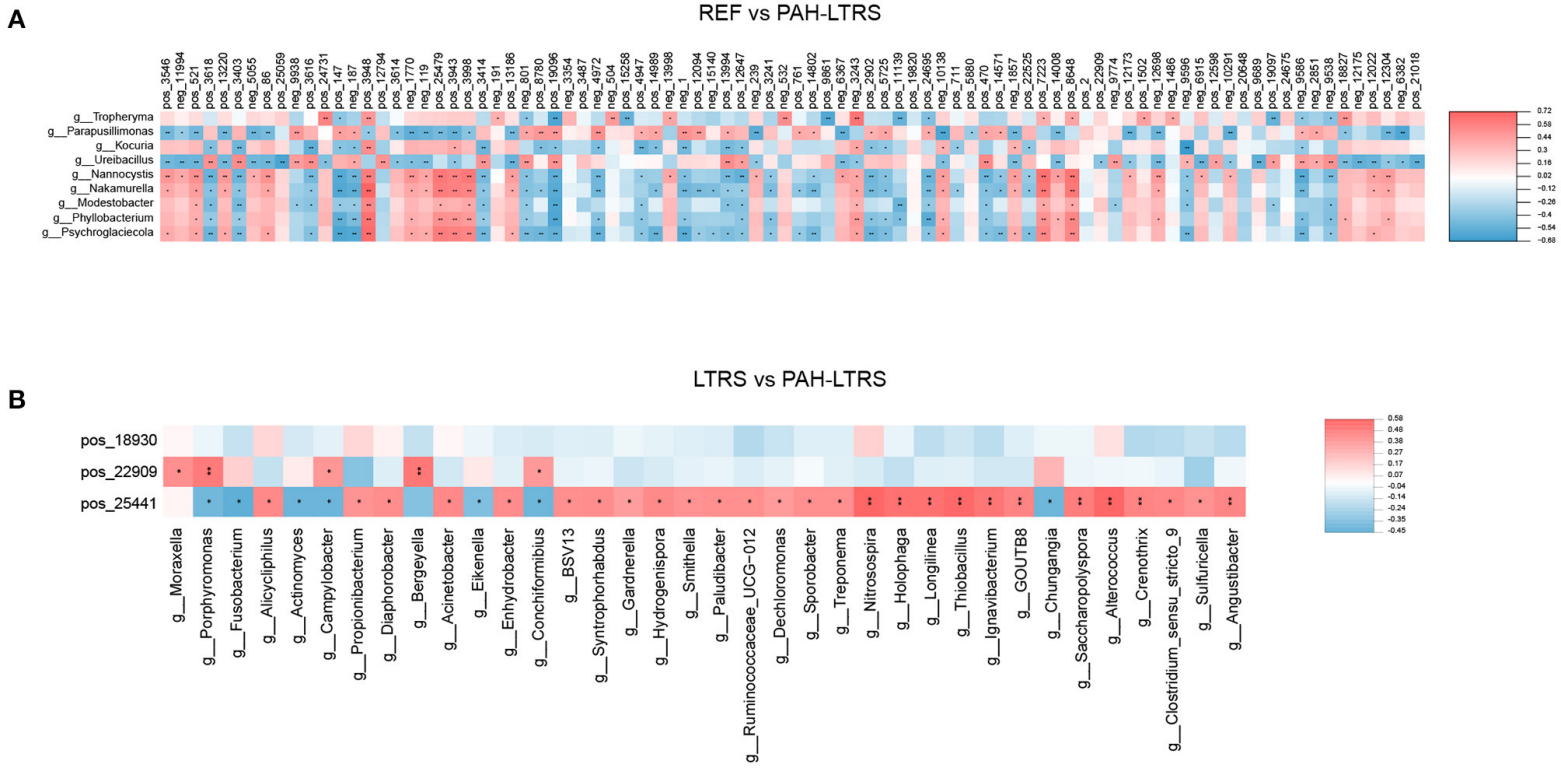
Significant correlation between lung bacteria and differential metabolites. Heatmap indicated positive (red) and negative (blue) correlations between metabolites and the microbiota at the genus level detected in REF compared with PAH-LTRS cohort **(A)** and LTRS compared with PAH-LTRS cohort **(B)**. The legend shows correlation values from −1 to 1. *Represents significantly negative or positive correlations (*P < 0.05, **P < 0.01). PAH-LTRS, patients with pulmonary arterial hypertension associated with Left-to-Right shunt (*n* = 19); REF, healthy reference (*n* = 20); LTRS, patients with Left-to-Right shunts but without PAH (*n* = 20).

We next analyzed the correlation between differential metabolites and microorganisms at the genus level. We found those differential metabolites were correlated with microbes at varying degrees (Supplementary Table 4). We focused on the significant positive or negative correlations between metabolites and microbial taxa (*p* < 0.05). Parapusillimonas was significantly enriched in **PAH-LTRS** (Figure 3A) and was positively correlated with differential metabolites, such as Uridine, neg_9938 (Glycoursodeoxycholic acid) and pos_19096 (Laudanosine), while negatively with Dimethylethanolamine. Compared with **REF**, UDP-glucose, pos_24731 (Guanidylic acid), Adenosine monophosphate, pos_18827 (2,4-DPD), and pos_12304 (Cinnamic acid) were significantly decreased in PAH-LTRS. These decreased metabolites were positively correlated with the genera, such as Tropheryma, and negatively correlated with the genera, such as Nannocystis (Figure 6A).

In the comparison of LTRS vs. PAH, DL-2-Aminooctanoic acid had no significant correlation with any differential bacteria, while Trigonellinamide and 2-piperidone were significantly correlated with related microbes. 2-piperidone was decreased considerably in PAH-LTRS compared with LTRS or REF (*p* = 0.005, 0.009, respectively), Integration analysis showed that 2-piperidone was significantly positively correlated with Moraxella, Porphyromonas, and Campylobacter, which with reduced abundance in PAH-LTRS (Figure 6B). Trigonellinamide was enriched in PAH-LTRS compared to LTRS (*P* = 0.001), and there was also a significant positive correlation with the genera enriched in PAH-LTRS, such as Alicycliphilus, Diaphorobacter, and Propionibacterium. Taken together, differential metabolites and their significantly correlated microbial taxa are expected to serve as diagnostic biomarkers and therapeutic targets for PAH-LTRS in children.

## Discussion

PAH is considered a severe disease associated with congenital Left-to-Right shunts heart disease (48, 49). Lung transplantation would be the only intervention for end-stage patients with PAH (18). Numerous studies on gut microbes in PAH, either in animal models or humans, elucidate the essential role of gut microbiota in this disease (32, 34, 35). However, there is a lack of information on the lung microbiota impact on the pathology and prognosis of PAH-LTRS. In the present work, we used multiple omics approaches to study the character of the lung microbiome, metabolome, and the correlation of them in children with PAH-LTRS.

In the current study, we found that there was no significant difference in alpha diversity among the three groups, indicating no significant differences globally in microbial communities, consistent with an earlier study of PAH in gut microbiota (34). In contrast, reduced lung microbial richness and diversity were found in other diseases (50, 51). PCoA, as the unsupervised analysis, could significantly detect the difference between groups. However, our PCoA results indicated that there was no significant clustering in Bray–Curtis distance matrices between groups. We assumed that there were individual differences among recruited participants and further studies with large cohorts size are needed to provide more evidence of global differences of lung microbes with PCoA clustering. PLS-DA, as the supervised method, could achieve clustering for different groups but with full awareness of the group labels (52–54). Composition analysis showed that there were bacterial disturbances in some taxa, despite the most common phyla and genera reported previously (39, 50) being the same among the three groups. Previous studies have shown that the Firmicutes (F) to Bacteroidetes (B) ratio (F/B), as the hallmark of gut dysbiosis (34, 35, 55), increased in the gut. F/B also tended to increase in PAH-LTRS (Supplementary Figure 1C). This ratio was mainly driven by less abundant Bacteroidetes phylum, propionate-producing bacteria and highly relevant in dysbiosis and disease (56–58). In contrast, there was no apparent change in the relative presence of Firmicutes phylum, which was consistent with previous findings in the gut (34). F/B was correlated with inflammation in gut microbiome studies, but the association of F/B with clinical significance in PAH-LTRS needs to be further addressed.

Lactobacillus, Alicycliphilus, Castellaniella, Propionibacterium, Providencia, Parapusillimonas, and Diaphorobacter genera were enriched in PAH-LTRS based on LEfSe analysis. In particular, Lactobacillus was thought to be reduced in PAH-LTRS in the gut (59). Lactobacillus in the lung was found to be significantly higher than in other groups and were the dominant taxa in PAH-LTRS. This discrepancy might result from different sub-type of PAH, tissue the microbes from, patient location, and the sampling time. The role of Lactobacillus in the lung of PAH-LTRS may be different from the gut. It might act as a beneficial bacterium as the short-term self-healing mechanism to resist PAH-LTRS development in the early stage. Further research needs to be done to explain the Lactobacillus variation in different studies and its role in PAH. Alicycliphilus phylum was considered involved in hypoxic energy metabolism involved in PAH, similar to the result of other studies concerning the lung bacteria (60), potentially as a marker of PAH-LTRS.

In the comparison of PAH-LTRS and REF, there were 88 differential metabolites disturbed. These metabolites were significantly enriched in a variety of metabolism pathways, including Galactose metabolism, Glycerophospholipid metabolism, Purine metabolism, Pyrimidine metabolism and Zeatin biosynthesis. Consistent with our result, Glycerophospholipid metabolism, Pyrimidine metabolism, and Purine metabolism were reported dysregulated in PAH (31, 61, 62). Glycerophospholipid metabolism was reported to be dysregulated in PAH by affecting lipid metabolism (63) and was close with inflammation (64), while inflammation is a prominent pathologic feature in PAH (65). However, there were only three different expressed metabolites in the PAH-LTRS cohort compared with LTRS, which exceeded our expectations. Such few differences may be due to the individual variability of the subjects and the insufficient sample size. 2-piperidone, which was significantly decreased in PAH-LTRS, was significantly positively correlated with Moraxella and Porphyromonas, which were decreased in abundance in PAH-LTRS. Porphyromonas was one of the most important genera of the phylum of Bacteroidetes and was reported involved in inflammatory diseases (66–68) and other human diseases (69, 70). Bacteroidetes might play a role in prohibiting PAH progression in patients with LTRS by Porphyromonas through related metabolites, such as 2-piperidone. 2-piperidone was reported to be disturbed in some human diseases, such as ovarian cancer (60), which indicated that it might be a biomarker for PAH-LTRS patients. Further evidence was warranted to clarify the mechanism of the significant correlation between the lung microbiota and metabolites.

However, our current study has some limitations. First, further study is required to clarify whether lung dysbiosis plays a pathophysiological role in the development of PAH-LTRS or is just secondary to pulmonary pathology resulting from the disease. If the former is true, these alterations might be helpful in investigating the underlying mechanisms of PAH-LTRS and open new avenues for therapeutic intervention. Second, further investigations consisting of larger sample sizes or samples from different centers or other geographical locations are needed in our future work. Third, BLAF samples were only collected from patients before surgery in this study, while samples from patients after surgery and collected at different time points after surgery will be more beneficial to predict or improve the clinical prognosis of PAH-LTRS. Fourth, BLAF collection was a method of invasive sampling. Instead, the upper airway method including oropharyngeal swabs and nasopharyngeal were much more convenient compared with BLAF collection (71, 72), and to what extent they can reflect the pathology of PAH-LTRS was worthy to study in the future.

In summary, our results demonstrate that bacterial composition and metabolic activity in lung were disturbed significantly in patients with PAH-LTRS. At the phylum level, Bacteroidetes was less abundant in PAH-LTRS. Lactobacillus, Alicycliphilus, Castellaniella, Propionibacterium, Providencia, Parapusillimonas, and Diaphorobacter were the predominant genus in PAH-LTRS. 2-piperidone and correlated microbes of Moraxella and Porphyromonas were significantly decreased in PAH-LTRS. Glycerophospholipid metabolism, Pyrimidine metabolism, and Purine metabolism were dysregulated in PAH-LTRS. Our study highlighted potentially unknown roles of pulmonary bacteria and metabolites in children with PAH-LTRS and might be the biomarkers providing a clue to improving the therapeutic, diagnostic, and clinical outcomes of PAH-LTRS.

## Data Availability Statement

The data presented in the study are deposited in the National Genomics Data Center (https://ngdc.cncb.ac.cn/omix/) repository, accession number OMIX001230.

## Ethics Statement

The studies involving human participants were reviewed and approved by Ethics Committee of Fuwai Yunnan Cardiovascular Hospital. Written informed consent to participate in this study was provided by the participants' legal guardian/next of kin.

## Author Contributions

RM and LC conceived and designed the experiments, wrote, revised, and finalized the manuscript. LC, YSo, YSu, WG, YD, CX, and ML performed the experiments and analyzed the data. All authors reviewed the content and approved the final version for publication.

## Funding

This work was supported by Foundation of Medical Specialist (YNWR-MY-2020-044) and Foundation Program of Yunnan Provincial Cardiovascular Clinical Medical Center (FZX2019-06-01-05).

## Conflict of Interest

The authors declare that the research was conducted in the absence of any commercial or financial relationships that could be construed as a potential conflict of interest.

## Publisher's Note

All claims expressed in this article are solely those of the authors and do not necessarily represent those of their affiliated organizations, or those of the publisher, the editors and the reviewers. Any product that may be evaluated in this article, or claim that may be made by its manufacturer, is not guaranteed or endorsed by the publisher.
