# Supplementary material for: Altered Lung Microbiome and Metabolome Profile in Children With Pulmonary Arterial Hypertension Associated With Congenital Heart Disease
Source: Front Med (Lausanne). 2022 Jul 28;9:940784. doi: 10.3389/fmed.2022.940784 (PMC9366172; doi:10.3389/fmed.2022.940784)
Supplement: Supplementary file 5 [file Data_Sheet_1.pdf]

Supplemental material

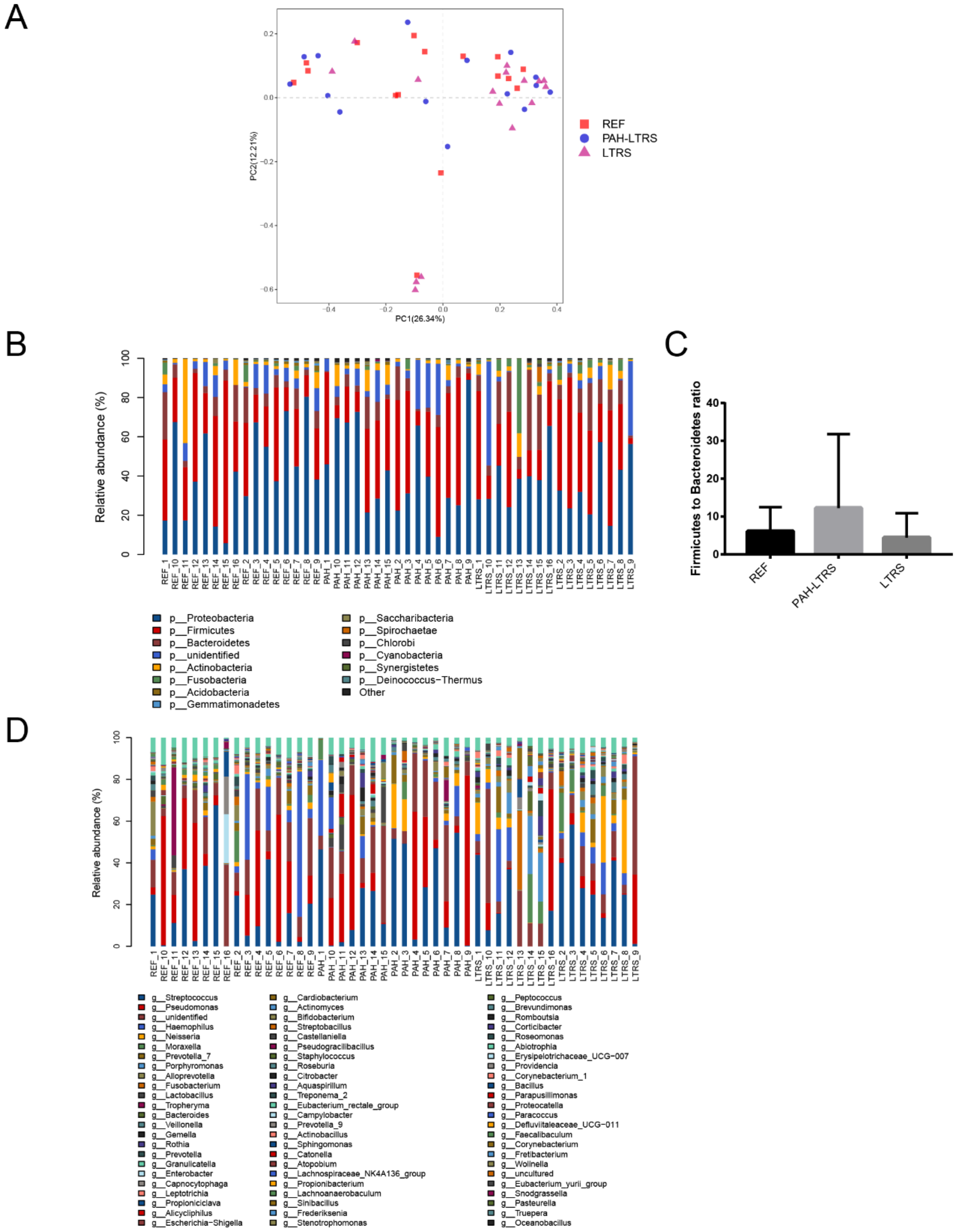

Supplementary Figure 1 PcoA plot and bacterial relative abundance of all samples. (A) Community similarity showed by the PcoA plot based on the bray-curtis distance. (B) Community composition in each sample at the phyla level. (C) F/B ratio

in different groups. (D) Community composition in each sample at the genus level. PAH-LTRS, patients with pulmonary arterial hypertension associated with Left-to-Right shunt (n=15); REF, healthy reference (n=16); LTRS, patients with Left-to-Right shunts but without PAH (n=16).

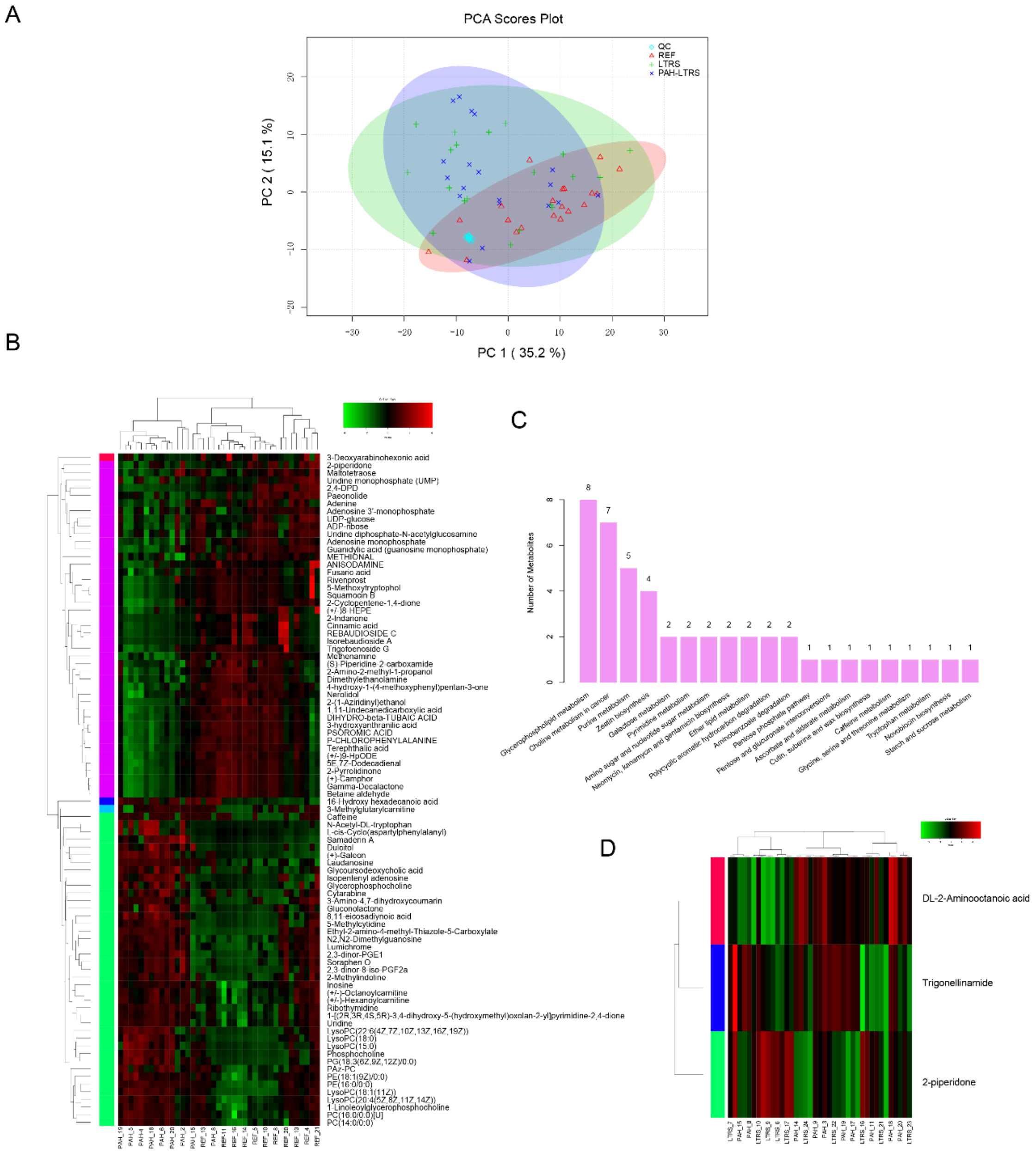

Supplementary Figure 2 PCA plot for all metabolome profiled samples including QC samples and clustering analysis of differential metabolites. (A) PCA was

utilized to confirm the LC-MS quality containing QC samples. (B) The differential metabolites in NAPH vs PAH-LTRS were clustered into 5 sub-clusters. (C) KEGG annotation in REF vs PAH-LTRS. (D) Heatmap of three differential metabolites in LTRS vs PAH-LTRS. PAH-LTRS, patients with pulmonary arterial hypertension associated with Left-to-Right shunt (n=19); REF, healthy reference(n=20); LTRS, patients with Left-to-Right shunts but without PAH (n=20).
